# Optimal voltage and frequency control strategy for renewable-dominated deregulated power network

**DOI:** 10.1038/s41598-024-84549-z

**Published:** 2025-01-02

**Authors:** Vineet Kumar, Sumit Sharma, Vineet Kumar, Sachin Sharma, Ark Dev

**Affiliations:** 1 Department of Electrical and Electronics Engineering, Madanapalle Institute of Technology and Science, Madanapalle, 517325 AP India; 2 KIET Group of Institutions Ghaziabad, Ghaziabad, India; 3 Parul Institute of Engineering and Technology, Parul University, Vadodara, Gujarat India; 4 Electrical and Electronics Engineering, Manipal Institute of Technology, Manipal Academy of Higher Education, Manipal, Karnataka India

**Keywords:** Stochastic generation, Random loading, Deregulated power system, Voltage regulation, Frequency management, Engineering, Electrical and electronic engineering

## Abstract

Maintaining stable voltage and frequency regulation is critical for modern power systems, particularly with the integration of renewable energy sources. This study proposes a coordinated control strategy for voltage and frequency in a deregulated power system comprising six Generation Companies (GENCOs) and six Distribution Companies (DISCOs). The system integrates thermal, diesel, wind, solar photovoltaic (PV), and hydroelectric sources. Two stochastic modeling techniques are used to characterize wind and solar generation, accounting for their variability within the control loops. A novel Leader Harris Hawks Optimization-based Model Predictive Controller (MPC-LHHO) is implemented, achieving a reduction in frequency deviation undershoot by 67.45% and voltage settling time by 91.11% compared to conventional controllers under poolco and bilateral transactions. Auxiliary devices, including the Unified Power Flow Controller (UPFC) and grid-connected electric vehicles (EVs), further enhance performance, reducing frequency deviations by 52.18% under stochastic scenarios. Rigorous evaluation under contract violations, random load variations, and renewable intermittency demonstrates the strategy’s robustness and efficacy.

## Introduction

The disparity between energy production and consumption can lead to several adverse events that impact the electrical grid and the equipment on both the generation and load sides. Ensuring the safe and reliable operation of any electrical system network requires maintaining the system frequency and terminal voltage within acceptable limits. Modern power networks are equipped with Automatic Voltage Regulators (AVR) and Load Frequency Control (LFC) mechanisms to address variations in voltage and frequency^1^. These mechanisms, along with additional regulatory schemes, provide the necessary control over frequency and terminal voltage in the power system. However, the increasing demand for renewable energy sources, along with rapid population growth and industrialization, has introduced new challenges in the field of automatic generation control (AGC) within power systems^2^. The advent of energy markets has significantly transformed global power system networks. In a deregulated power system, multiple Generation Companies (GENCOs) and Distribution Companies (DISCOs) participate in a competitive electricity market overseen by a central entity known as the Independent System Operator (ISO)^3^.

Previously, researchers have addressed the automatic generation control (AGC) problem in traditional settings using classical controllers^4–7^. The interest in meta-heuristic-based PID controllers for studying LFC issues in deregulated environments has grown significantly^8^. Additionally, different fractional and hybrid versions of PID controllers, such as FOPID, TID, and PIDF (1 + FOD) controllers, have been previously employed in AGC studies^9–12^. The integration of renewable resources has received significant attention in research concerning LFC, both in classical and deregulated settings using classical controllers. Sharma et al.^13^ explored the challenges of frequency regulation in a deregulated power system with a fuzzy-based regulator combined with a redox flow battery^14^. In recent years, battery storage-based auxiliary frequency control methods have gained considerable importance^15^. Superconducting Magnetic Energy Storage (SMES) devices have also attracted attention for additional frequency regulation^16,17^. Due to the high cost of storage devices, various researchers have proposed more affordable alternatives, such as dynamic energy storage technologies. These include the integration of electric vehicles with the grid via vehicle-to-grid (V2G) technology, dynamic demand response, and controllable loads^18–20^. The concept of virtual inertia has been utilized to prevent unexpected frequency dips caused by fluctuating loads^21^. To further reduce power system frequency oscillations, several FACTS devices, such as Thyristor Controlled Series Capacitor (TCSC), Thyristor Controlled Phase Shifter (TCPS), and Interline Power Flow Coordinator (IPFC), have been integrated with controlled LFC systems^22^. Most work on simultaneous voltage and frequency control has been conducted in vertically integrated systems^23,24^. In the deregulated wind-integrated power system, Rajbongshi et al. proposed a proportional plus fractional integral plus derivative with filter (PI^λ^DF) controller for combined AVR-LFC loops, though the voltage and frequency oscillations took a considerable time to settle^25^. Only a few studies have addressed this issue with solar and wind integration, with major focus on vertically integrated power systems for optimal control of combined AVR and LFC loops using classical as well as advanced controllers^26–30^.

A comprehensive review of the literature on combined voltage and frequency control indicates a major reliance on classical controllers, including various versions of PI, PID, and FOPID controllers. Therefore, the combined AVR-LFC control problem in deregulated power systems warrants further research with advanced predictive controllers^31^. Moreover, studies on voltage and frequency control in deregulated environments have often overlooked the stochastic nature of renewable generation. Furthermore, some recent studies are reviewed in the field of voltage and frequency regulation^32–35^.

Several recent works have integrated renewable sources with classical control mechanisms. For example, Rajbongshi et al. proposed a proportional-plus-fractional-integral-plus-derivative (PIλDF) controller for combined AVR-LFC loops, but the resulting system exhibited slow settlement of frequency and voltage oscillation studies have used advanced controllers such as the fuzzy-based regulator with redox flow batteries, but the literature remains limited to vertically integrated systems, leaving the deregulated power market relatively unexplored. Additionally, the role of auxiliary control devices like Unified Power Flow Controllers (UPFC) and Electric Vehicles (EVs) has been considered in some studies, but these devices are often analyzed in isolation, without a comprehensive strategy that integrates them with advanced predictive control methods.

In this study, a deregulated multi-area, multi-source power network has been studied for the combined voltage and frequency control problem for different deregulated market cases, such as poolco transactions and bilateral transactions. An optimal MPC-LHHO controller has been designed by the authors in^29^ for the centralized control of AVR and LFC loops in a vertically integrated power network. This work addresses the research gap in deregulated power systems by proposing a novel coordinated voltage and frequency control strategy that integrates advanced Leader Harris Hawks Optimization-based Model Predictive Control (MPC-LHHO) with stochastic modeling of wind and solar generation. Unlike traditional approaches, the proposed method incorporates stochastic models for renewable energy generation based on ARIMA, allowing for more accurate prediction of power fluctuations and enhanced system stability. Furthermore, the study integrates UPFC and EVs as auxiliary control devices, presenting a holistic control strategy for frequency regulation under variable renewable conditions in a deregulated market.

The novelty of this work lies in the novel combination of MPC-LHHO with stochastic renewable generation models and the simultaneous optimization of multiple control loops (AVR, LFC, and auxiliary control devices) in a deregulated power system. This is the first study to address the dual challenge of voltage and frequency regulation while accounting for contract breaches, random load variations, and the complex interactions in a deregulated environment, showcasing significant improvements in system stability and robustness^36^. The leader-based mutation selection has further amplified its performance by enhancing the exploration and exploitation phases^37^. Herein, the LHHO-optimized MPC has been implemented on the concerned deregulated system, and its transient response qualities have been examined and compared with the HHO-based Model Predictive controller as well as the conventional PID-FA^27^ and FOPID-MFO^28^ controllers. The sudden oscillation in the frequency and tie-line power flow that was caused by the intermittent nature of the load and the generation from renewable sources has been mitigated using the utilization of a Unified Power Flow Coordinator and the integration of Electric Vehicles (EVs) with the grid. These auxiliary methods of control have been deployed in conjunction with the MPC controller, and the controller weights have also been obtained using LHHO in coordination with EV and UPFC loops. The major novelties and highlights of this work are given as,

1. A novel approach is proposed for the simultaneous control of voltage and frequency deviations within a deregulated power system that integrates wind, photovoltaic (PV), and small hydro generation sources, employing the Leader Harris Hawks Optimized Model Predictive Controller (MPC-LHHO). The transient response of the MPC-LHHO is analyzed and compared to that of established controllers, including the PID-FA^27^, FOPID-MFO^28^, and MPC-HHO^36^, across various scenarios, such as poolco and bilateral transaction frameworks.
2. Further in a novel attempt, the randomness in wind and solar generation has been considered using two different stochastic models (SM-1 and SM-2). Under SM-1 the small stochastic drifts and deterministic drifts were modelled using a mathematical function. While in SM-2, the stochasticity in RESs has been modelled after using the Monte Carlo simulations of the ARIMA model constructed for real-time wind speed and solar irradiance data procured from NREL.
3. To deal with intermittencies in load and renewable sources, the authors have proposed the application of a Unified Power Flow Coordinator and Electric Vehicle integration with the grid. The simulation results have verified that a significant reduction has been recorded in the frequency fluctuations of the system.
4. The validation of robustness has been obtained after considering the contract violation case and randomly varying load pattern in the UPFC-EV-based MPC-LHHO controlled combined AVR-LFC model.

Section “Introduction” presents the basic introduction and literature review of the frequency and voltage control problem in interconnected power system networks under deregulated environment. The brief modelling of the combined AVR-LFC model along with UPFC and EV blocks has been discussed in “System modelling”. Section “LHHO-based model predictive control scheme” illustrates the LHHO-based optimal MPC strategy for the simultaneous control of voltage and frequency. Section “Results and discussion” presents the important findings and discussion related to the proposed work, followed by concluding remarks in the “Conclusions” section.

## System modelling

### Model for basic transient response analysis

**Fig. 1:**
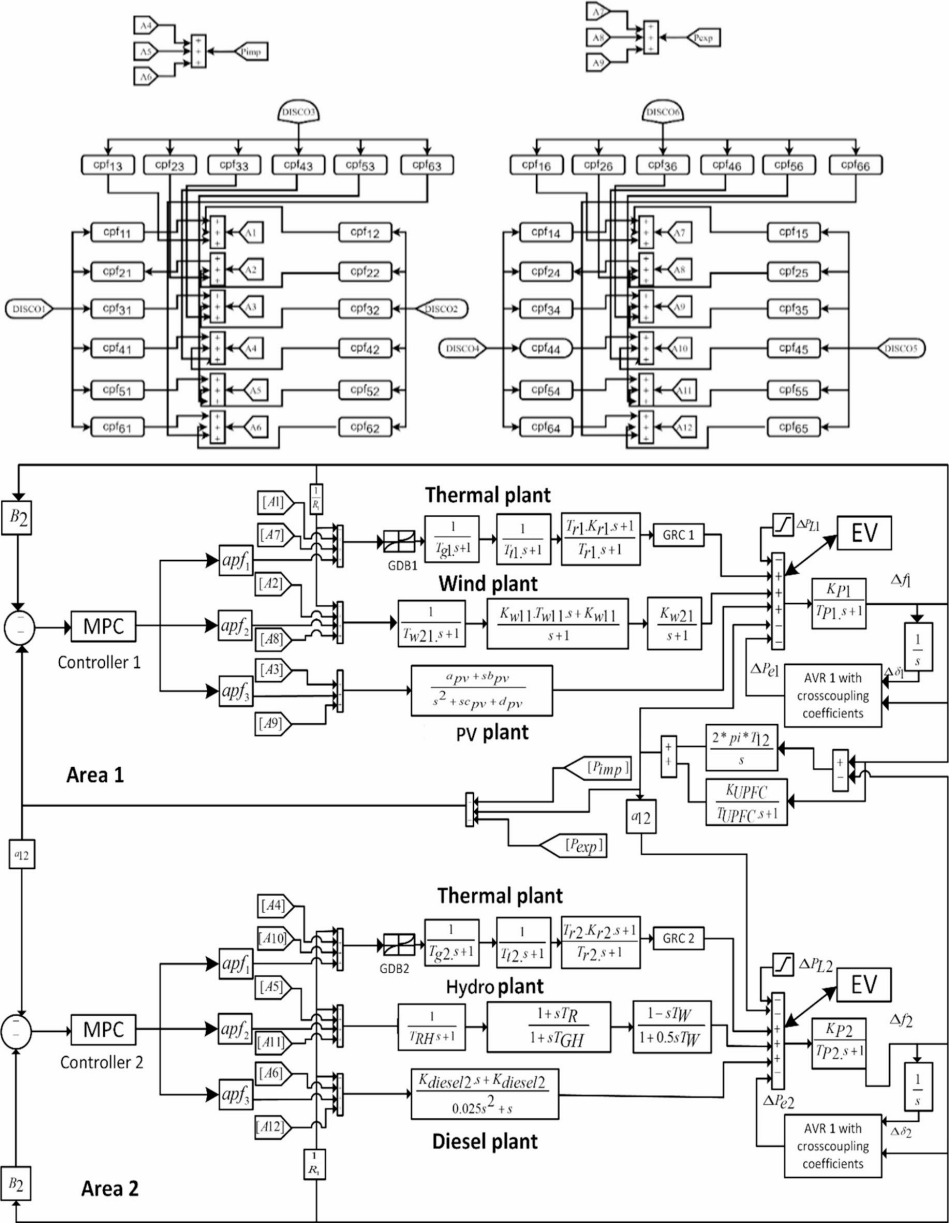
Combined AVR-LFC model for the considered power system model under deregulated environment.

This study analyses a combined AVR-LFC model containing 6 GENCOs and 6 DISCOs as depicted in Fig. 1. Herein, renewable power resources such as wind, solar PV, and hydro have been considered in integration with the conventional thermal and diesel generation systems. Area 1 GENCOs contain thermal, wind, and solar PV plants, and area 2 GENCOs contain thermal, diesel, and hydro plants. The generation capacity of area 2 is four times that of area 1. In this research work, authors have proposed 3 different methods for the modeling of solar and wind generation systems under a deregulated environment. Basic transient analysis features transfer function-based modeling wherein the stochastic nature of wind and solar has not been studied. In stochastic modelling (SM-1), the stochastic drifts and deterministic shifts have been considered for the modelling of wind and solar generation output values. In another case (SM-2), practical wind speed and solar irradiance data have been considered and stochasticity has been added using Monte Carlo simulations. In Fig. 1, the MATLAB SIMULINK based modelling for the combined AVR-LFC loops has been referred from the previously published works in the conventional and deregulated environment^25,29^. Detailed transfer function-based modelling for thermal, solar PV, wind, and hydro are provided in^30,38,39^. Additionally, the DISCO participation matrices (DPMs) and area participation factors (apfs) corresponding to the deregulated modelling under poolco, and bilateral cases are provided in Sect. 4.1.

This work also features the application of several auxiliary control loops for the improvement of frequency stability in the power system model. Herein, Unified Power Flow Coordinator (UPFC) and Electric Vehicle (EV) integration with the grid has been considered to provide additional frequency support in the system in coordination with the proposed MPC-LHHO scheme. From the literature, it has been established that these auxiliary loops do not have an impact on the voltage and tie-line power flow deviations in the system. However, these auxiliary loops significantly improve the frequency profile of the system. In this work, several means of auxiliary controls have been adopted to curb the frequency oscillations caused due to the variations in the consumer load and renewable generation. Herein, the Unified Power Flow Controller has been employed to get improvements in the frequency regulation by providing fast-acting reactive power compensations in the tie-line. Such fast compensation will ensure a smooth lossless flow of power among different neighboring areas, and it will improve the damping of the system and power angle stability. The block diagram and detailed modelling of a UPFC device connected to a power system model may be referred to from^7^.

**Fig. 2:**
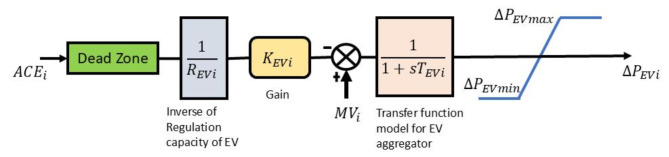
Block diagram representation for electric vehicle model.

Recently, the concept of electric vehicle integration with the grid has gained much interest from various researchers working in the field of power structure management and regulation^7^. The electric vehicle unit in this work is basically an array of electric vehicles connected to the grid using V2G technology. These vehicles act as dynamic storage depending on the energy demand from the consumer side. During high demand, the vehicle battery will be used to supply power to the grid, and in case of reduced demand, the grid will supply excess power to the battery charging station. The change in output power ($\:{\varDelta\:P}_{EVi}$) of EV is a function of EV gain $\:{(K}_{EVi}$) and regulation capacity of EV ($\:{R}_{EVi}$). The up and down limits on the yield power of EV are considered as ± 5 kW. Figure 2 explains a basic method for the interconnection of EV aggregation with the grid^38,40^.

### Stochastic modelling of solar and wind units

**Fig. 3:**
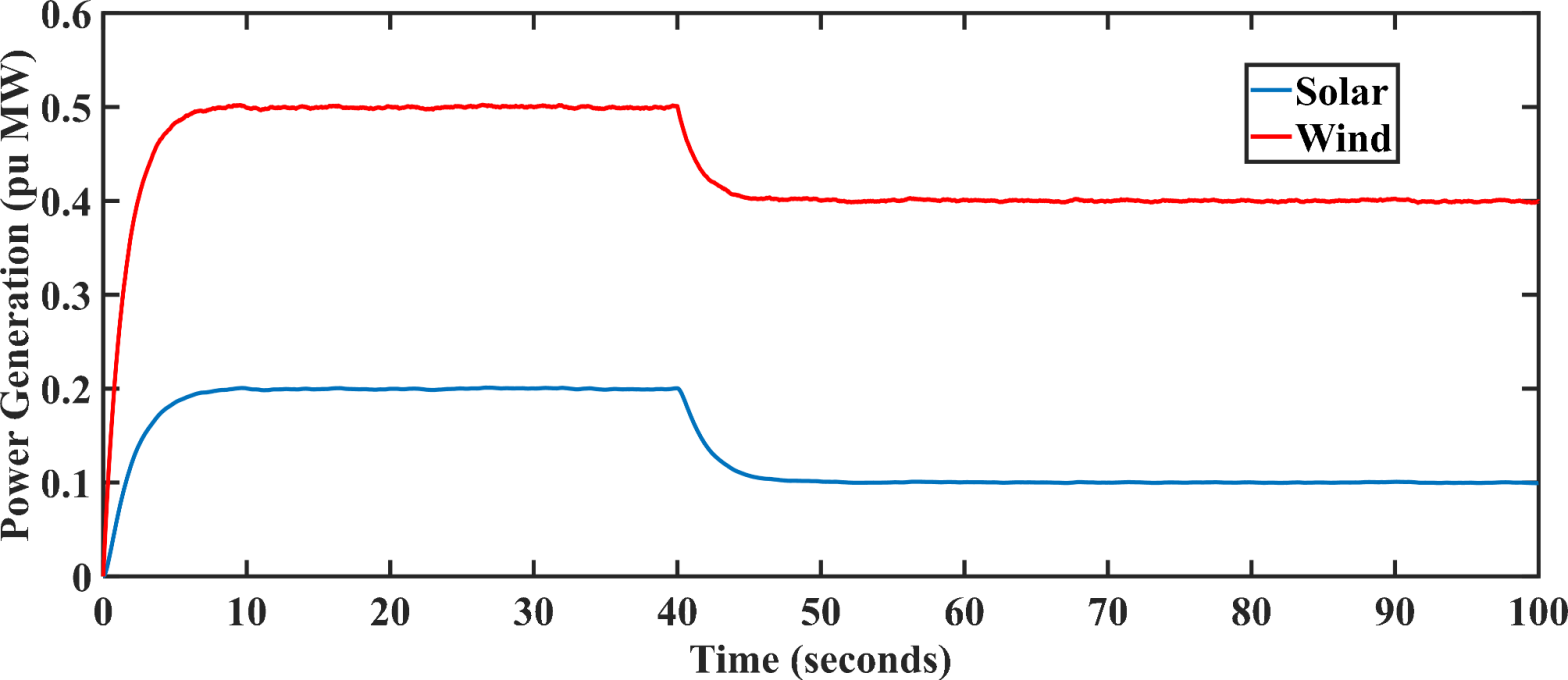
Fluctuations in generated solar and wind power output with respect to time for SM-1^29,41^.

Herein, the LFC model in Fig. 1 has been integrated with additional solar PV and wind generation units containing a randomly varying pattern comprising both deterministic shifts and stochastic drifts. This particular model (SM-1) has been previously used in the LFC model^41^ and combined AVR-LFC model for conventional power system models^29^. Herein, the net generated power from wind and solar is calculated using a mathematical model given in Eq. (1). And the graphical representation for the output wind and solar power is given in Fig. 3.

1 $$\:P\left(g\right)=\left[\right\{\varphi\:\eta\:\sqrt{\beta\:}(1-G(s\left)\right)+\beta\:\}\partial\:/\beta\:]\varGamma\:$$

*P*(*g*) is the total generation power from both solar and wind plants and is depicted in Fig. 3. Herein, $\:\varphi\:$ is the stochastic element having random values from − 1 to + 1, and for both cases, $\:G\left(s\right)=\frac{1}{1+1{0}^{4}s}$. And $\:\varGamma\:$is specified as *0.5 H*(*t*)*-0.1 H*(*t-40*) for wind turbine model and *1.111 H*(*t*)*-0.5555 H*(*t-40*) for solar PV model.

**Fig. 4:**
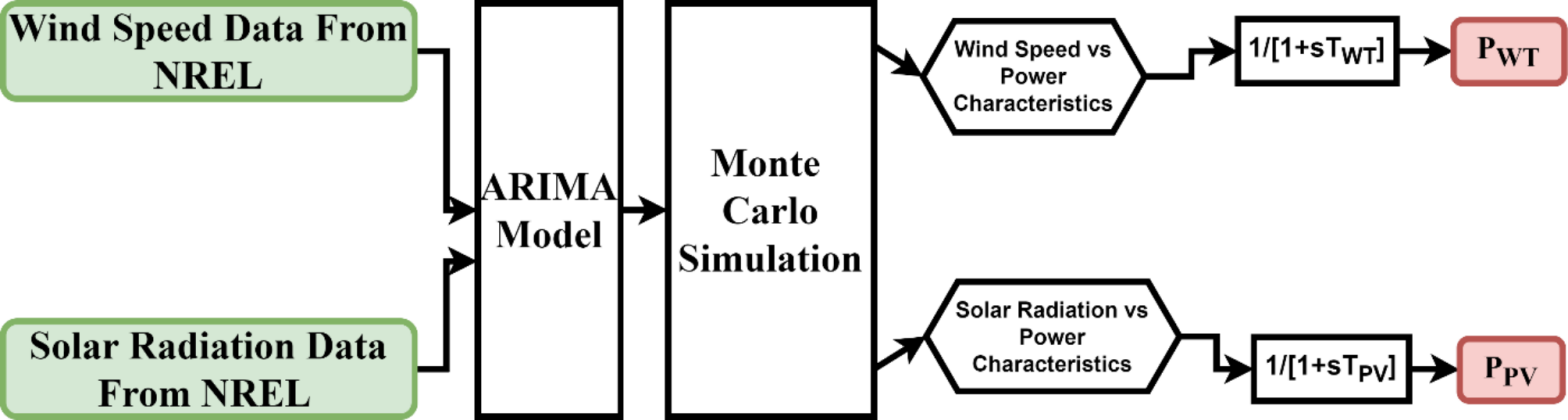
Proposed strategy for the modelling of wind and solar generation under SM-2.

**Fig. 5:**
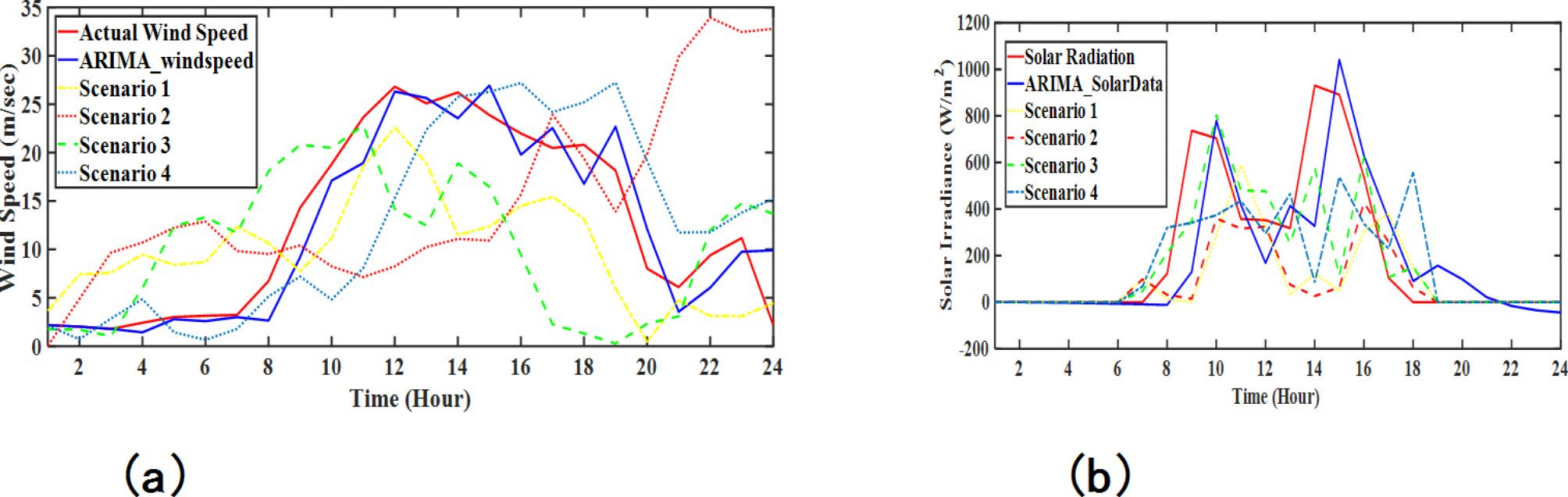
Different scenarios generated using ARIMA models for: (**a**) wind speed, (**b**) solar irradiance.

In another stochastic model (SM-2), the stochasticity in wind speed and solar radiation data has been introduced after obtaining the ARIMA model of real-time data taken from the National Renewable Energy Laboratory (NREL)^42^. Here, an ARIMA (2,1,3) model has been used to model the wind speed data and ARIMA (2,1,2) has been used to model the solar irradiance data. All the modelling coefficients are given in Table 1, and the resulting ARIMA models have been plotted to show a comparison with the actual data. The flowchart for the SM-2 modelling strategy is given in Fig. 4, and the resulting wind and solar data scenarios are given in Fig. 5. These ARIMA models are then simulated to generate 4 different scenarios of wind speed and solar irradiance. These generated scenarios for 24-h are sampled in seconds, and 100 data samples for each scenario are saved for further processing. Further, for wind speed data samples, the typical speed vs. power characteristic was used to calculate the net generated wind turbine power^38,43^. Further, for solar radiation data, the output power characteristics given by Shimizu et al., Matsuo et al., and Nakagawa et al.^32^ have been employed to calculate the net solar PV power. The extracted power from the PV cell is determined using Eq. (2).

2 $$\:{P}_{PV}=\:\eta\:S\varphi\:\:\left(1-0.005\left({T}_{a}+25\right)\right)$$

Here, $\:\eta\:$ is the efficacy of PV, *S* is the PV surface area,$\:\:\varphi\:$ is the intensity of Solar radiation, and $\:{\:T}_{a}$ is the surrounding temperature.

In this study, the ARIMA (Auto Regressive Integrated Moving Average) model is utilized to generate stochastic scenarios for wind and solar power generation. The ARIMA model is well-suited for forecasting time series data with inherent randomness and trends, such as wind speed and solar irradiance. To model these parameters, MATLAB’s Econometric Modeler was employed, which provides an efficient environment for fitting ARIMA models to real-time wind and solar data obtained from the National Renewable Energy Laboratory (NREL). For the wind speed data, an ARIMA (2,1,3) model was developed, and for the solar irradiance data, an ARIMA (2,1,2) model was chosen. The ARIMA parameters for both wind speed and solar irradiance were estimated using MATLAB’s Econometric Modeler, which applies Maximum Likelihood Estimation (MLE) to find the optimal values for the AR and MA terms.

The econometric tool also enabled the validation of the ARIMA model by comparing the generated time series against real-time data from NREL. This validation ensured that the model parameters (AR and MA coefficients) accurately reflected the stochastic nature of wind and solar power generation.

**Table 1:** Coefficients for ARIMA model considering wind and solar data.

Coefficients	ARIMA (2,1,3) for wind speed	ARIMA (2,1,2) for solar radiation
AR	[1.15931546777504; − 0.546787232455465]	[0.698892516265124; − 0.808177213995103]
MA	[− 0.405647930586124; − 0.508668957533235; 0.896978642790202]	[− 0.519638262169804; 0.455910957719170]

## LHHO-based model predictive control scheme

In this article, a robust centralized Model Predictive Controller (MPC) is examined and implemented to regulate both frequency and voltage within a hybrid energy system in a deregulated environment. This advanced predictive controller leverages a novel meta-heuristic optimization method, enhancing the system’s transient profile. The standout feature of this work is the optimization of MPC cost characteristic weights using the Leader Harris Hawks Optimization (LHHO) method. While MPC is a reliable technique for various dynamical systems, its effectiveness is notably improved by fine-tuning the cost function weights, particularly for managing multi-loop dynamics. The authors introduce a meta-heuristic optimization strategy designed to precisely adjust the controller weight values. This article explores the essential components and steps of the LHHO-MPC approach, providing a thorough analysis of how this innovative strategy effectively tackles the complex combined voltage and frequency issues in a deregulated power network. The strategic integration of the MPC framework with the adaptive LHHO optimization method represents a novel approach to ensuring the stability and efficiency of hybrid energy systems in a dynamic deregulated environment.

1. Model the concurrent voltage and frequency regulation loops for the deregulated power structure on the SIMULINK platform.
2. Employ MPC using the design tool and supply the values for Measured Outputs (MOs), Manipulated Variables (MV), sampling time, prediction horizon, and control horizon.
  Here, area control error values in both areas are taken as MVs (input variables), and frequency deviation is taken as MOs (output variables) corresponding to LFC loops. Similarly, the input signal to the exciter is taken as MVs and terminal voltage output is taken as MOs for AVR loops in both areas.
3. Select the weights on input, output, and rate of change of input using online features in MPC design and provide nominal values for chosen weights and run the model.
4. Execute LHHO method for the optimum tuning of the above-mentioned controller weights. The tuning of MPC weights should be performed in such a manner that the Performance Index (PI) presented in Eq. (3) is minimized.
  3 $$\:\text{P}\text{I}\:=\:{(\alpha\:.ISE}_{1})+{(\beta\:.ISE}_{2})$$
  where, $$\:{ISE}_{1}={\int\:}_{0}^{T}\left\{{(\varDelta\:{f}_{i})}^{2}+{(\varDelta\:{T}_{pij})}^{2}\right\}dt\:and\:{ISE}_{2}={\int\:}_{0}^{T}\left\{{\varDelta\:{V}_{i}}^{2}\right\}$$dt.
  Here, $\:\varDelta\:{f}_{i}$ and $\:\varDelta\:{V}_{i}$ are the frequency error and voltage error values in *i*^th^ area, and $\:\varDelta\:{T}_{pij}$ is the tie-line power error between area *i* and *j*. $\:{ISE}_{1}$ and $\:{ISE}_{2}$ are the values of integral squared errors associated with the LFC and AVR loops, respectively.
5. Choose the suitable values for ${\alpha}\:\text{and}\:{\beta}$ to get the required performance.
  The flow diagram for the proposed LHHO-based MPC method is provided in Fig. 6. Further, a detailed description of the MPC and LHHO approaches is provided in the subsequent sections.
  **Fig. 6:**
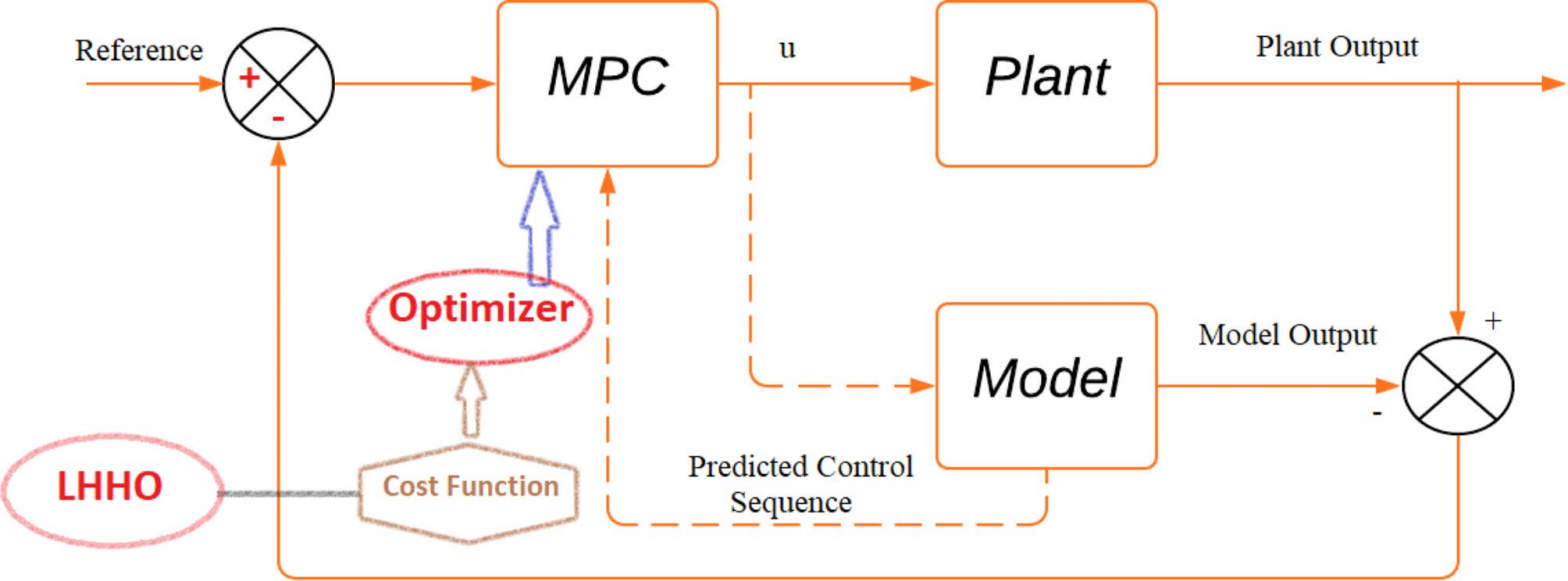
Block diagram for the proposed MPC-LHHO scheme.

### Model predictive control

As opposed to traditional PID and MPC controllers, the centralized MPC algorithm proposed here better coordinates the voltage and frequency management loops and effectively deals with their interface. Since MPC can process several inputs and outputs simultaneously, it is an efficient control method for complicated systems with linear and non-linear dynamics. Since nonlinearities, uncertainties, and external disturbances are inherently present in any power system model, MPC is essential in order to compensate for them adequately. The prediction unit and the control unit are the two main components of an MPC block. At the current instant of time, where the past data on the inputs/outputs and array of control sequences are available for a specific prediction horizon, the prediction unit is responsible for making predictions on plant output. Control computations are then carried out optimally by minimizing a cost function in Eq. (4), and the control unit determines future control input by analyzing past input/output data and forecasts of future output.

4 $$\:\varvec{J}=\:{\sum\:}_{\varvec{h}={\varvec{H}}_{1}}^{{\varvec{H}}_{2}}\varvec{o}1{{[\varvec{E}}_{\varvec{p}}(\varvec{t}+\varvec{h})]}^{2}+{\sum\:}_{\varvec{m}=1}^{\varvec{M}}\varvec{c}1[{\varDelta\:\varvec{U}(\varvec{t}+\varvec{m}-1)]}^{2}$$

Here, H_1_ and H_2_ are bounds on the set of predicted samples, M is a bound on the control sequence, E_p_ is the prediction error between the reference output and the predicted output values, and U is the incremental control input sequence. The optimizer will solve this cost function (CF) for each time sample to get its optimal control sequence values. Figure 6 presents the fundamental architecture for MPC tuned using the LHHO algorithm.

In this problem, the Model Predictive Controller (MPC) addresses a Multiple Input Multiple Output (MIMO) system with 4 input and 4 output variables. The prediction horizon is set to 10, and the control horizon is 2. The dataset for the MPC cost function includes CF weights o1 and c1, representing the error in anticipated output estimations and the variance between adjacent input samples, respectively. These weights determine the cost associated with each input and error value. The controller intervenes to prevent the output from deviating from the set point if the weight corresponding to any projected error is large. Similarly, the MPC ensures that no input deviates from its starting value if the coefficient for that input is large. A penalty on the rate of change in control can be applied to penalize the difference between current and previous input values. An additional weight (dc1) is used to impose a penalty on the incremental change of the input vector. By appropriately adjusting the cost function weights, the MPC controller can manage multi-loop problems and various objectives. These weights can range between zero and one and can be optimized using a meta-heuristic algorithm to achieve the desired outcomes^29,31^.

### Leader HHO algorithm

Harris Hawks Optimization (HHO) is a nature-inspired optimization technique that mimics the chasing and hunting behavior of Harris Hawks. The optimal solution is represented by the position of the Jackrabbit, with each Hawk in the group representing a potential solution to the problem. HHO excels in optimization due to its well-defined exploration and exploitation phases, along with exceptional convergence abilities. The procedural steps of HHO are detailed in the literature, specifically in reference^36^. To further enhance the HHO algorithm, some modifications have been proposed in^37^. The introduction of an adaptive perch probability feature, which depends on the positions of the worst- and best-performing Hawks, has significantly improved the algorithm’s search process. Additionally, a leader-based mutation selection method has been implemented to simulate the transition from exploration to exploitation. This feature greatly enhances the Hawks’ search capabilities, resulting in the advanced Leader Harris Hawks Optimization (LHHO). The pseudocode and algorithmic steps for this approach can be found in reference^37^.

The Leader Harris Hawks Optimization (LHHO) approach used in this study introduces significant advancements by enhancing the exploration and exploitation phases of the traditional HHO algorithm. The adaptive perch probability and leader-based mutation selection improve convergence speed and ensure global optimization, making it particularly effective for complex multi-loop control problems in power systems. Future work could explore extending the LHHO framework to hybrid optimization techniques or applying it to other domains, such as energy management in microgrids or large-scale renewable integration scenarios, to further validate its versatility and performance.

**Table 2:** Parameter values for different controllers.

Transaction	Parameters			
Controllers	PID-FA^27^	FOPID-MFO^28^	MPC-HHO^29^	MPC-LHHO
Poolco transaction				
Area 1 (LFC)	K_p_ = 2.9841; K_i_ = 3; K_d_ = 2.9855	K_p_ = 3; K_i_ = 3; K_d_ = 2.9986; la = 1; mu = 0.4893	o1 = 0.8467; c1 = 0.0806; dc1 = 0.0051	o1 = 0.9922; c1 = 0.0806; dc1 = 0.0031
Area 1 (AVR)	K_p_ = 0.2746; K_i_ = 0.0638; K_d_ = 0.0646	K_p_ = 0.1014; K_i_ = 0.1664; K_d_ = 0.0728; la = 0.9326; mu = 0.8131	o1 = 0.2565; c1 = 2.16 × 10^−8^; dc1 = 2.92 × 10^−19^	o1 = 0.3278; c1 = 2.1 × 10^−8^; dc1 = 0.0200
Area 2 (LFC)	K_p_ = 2.9957; K_i_ = 2.9886; K_d_ = 2.983	K_p_ = 2.9992; K_i_ = 2.9982; K_d_ = 3; la = 0.9977; mu = 2.43 × 10^−6^	o1 = 0.6967; c1 = 1.5 × 10^−6^; dc1 = 0.0020	o1 = 0.9921; c1 = 1.41 × 10^−6^; dc1 = 0.0599
Area 2 (AVR)	K_p_ = 0.1849; K_i_ = 0.0827; K_d_ = 0.1984	K_p_ = 0.0963; K_i_ = 0.1231; K_d_ = 0.2587; la = 1; mu = 0.5932	o1 = 0.1029; c1 = 2.56 × 10^−10^; dc1 = 0.0564	o1 = 0.1029; c1 = 1.07 × 10^−13^; dc1 = 0.0804
Bilateral transaction				
Area 1 (LFC)	K_p_ = 2.9447; K_i_ = 2.448; K_d_ = 2.9448	K_p_ = 3; K_i_ = 2.9999; K_d_ = 3; la = 1; mu = 0.9481	o1 = 0.8947; c1 = 0.0013; dc1 = 0.0566	o1 = 1; c1 = 0.0016; dc1 = 0.0096
Area 1 (AVR)	K_p_ = 0.067; K_i_ = 0.0366; K_d_ = 0.0716	K_p_ = 0.8698; K_i_ = 0.1575; K_d_ = 2.3165; la = 1; mu = 1	o1 = 0.2857; c1 = 0.0034; dc1 = 0.1669	o1 = 0.3973; c1 = 0.0345; dc1 = 0.1540
Area 2 (LFC)	K_p_ = 2.9447; K_i_ = 2.945; K_d_ = 2.9448	K_p_ = 3; K_i_ = 2.999; K_d_ = 3; la = 0.9998; mu = 0.3394	o1 = 0.8654; c1 = 2.1 × 10^−9^; dc1 = 0.5645	o1 = 1; c1 = 0.0345; dc1 = 0.0485
Area 2 (AVR)	K_p_ = 2.9448; K_i_ = 1.1112; K_d_ = 2.945	K_p_ = 1.0947; K_i_ = 0.1217; K_d_ = 2.7131; la = 1 = 0.9999; mu = 0.9894	o1 = 0.5287; c1 = 0.00003; dc1 = 0.1581	o1 = 0.4757; c1 = 0.0691; dc1 = 0.0223

## Results and discussion

### Basic transient performance

**Fig. 7:**
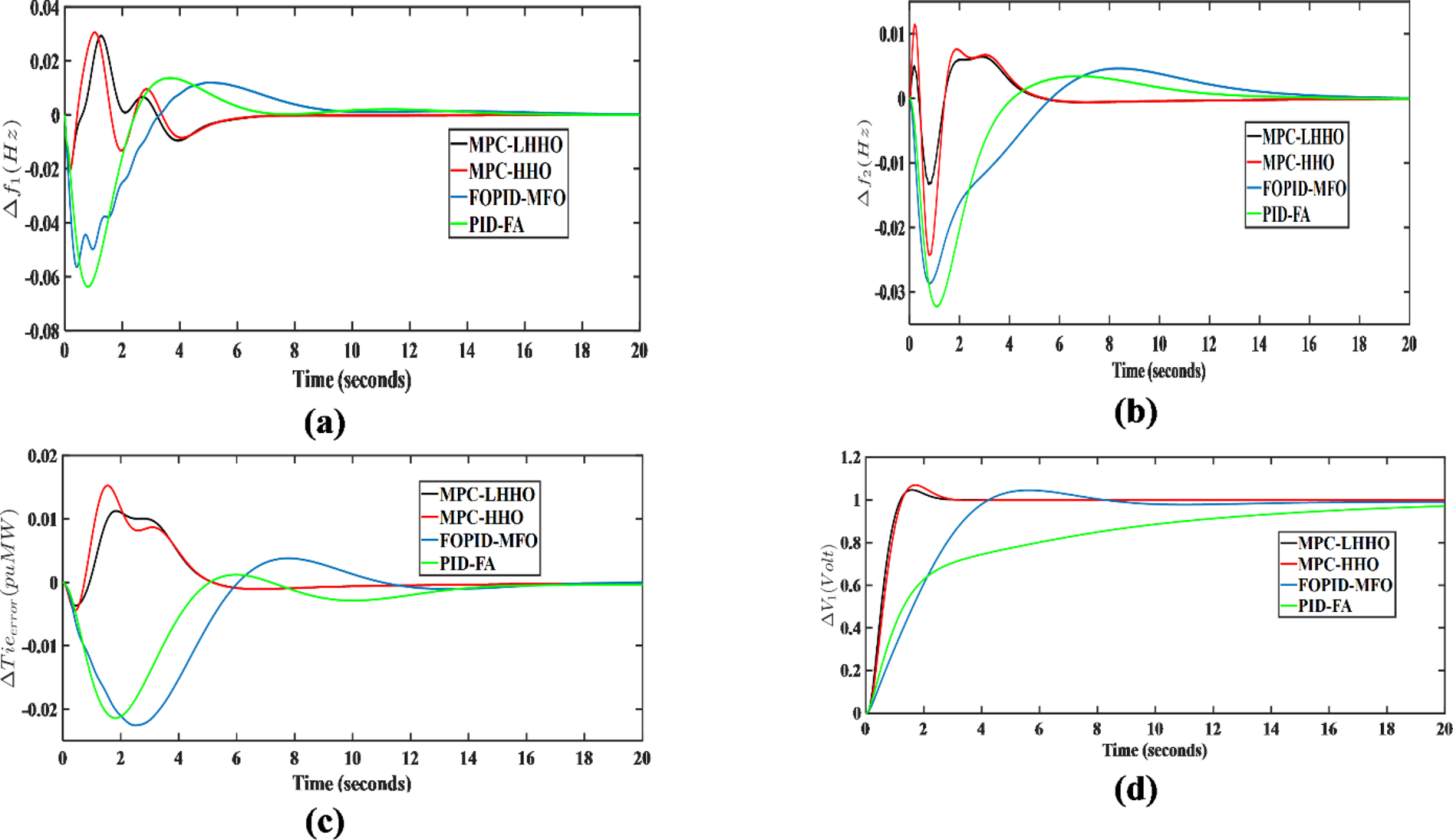
Transient response curves for poolco transaction case considering; (**a**) $\:\varDelta\:{f}_{1}$ (Hz), (**b**) $\:\varDelta\:{f}_{2}$ (Hz), (**c**) $\:\varDelta\:{T}_{p12}$ (puMW), (**d**) $\:\varDelta\:{V}_{1}$ (V).

**Fig. 8:**
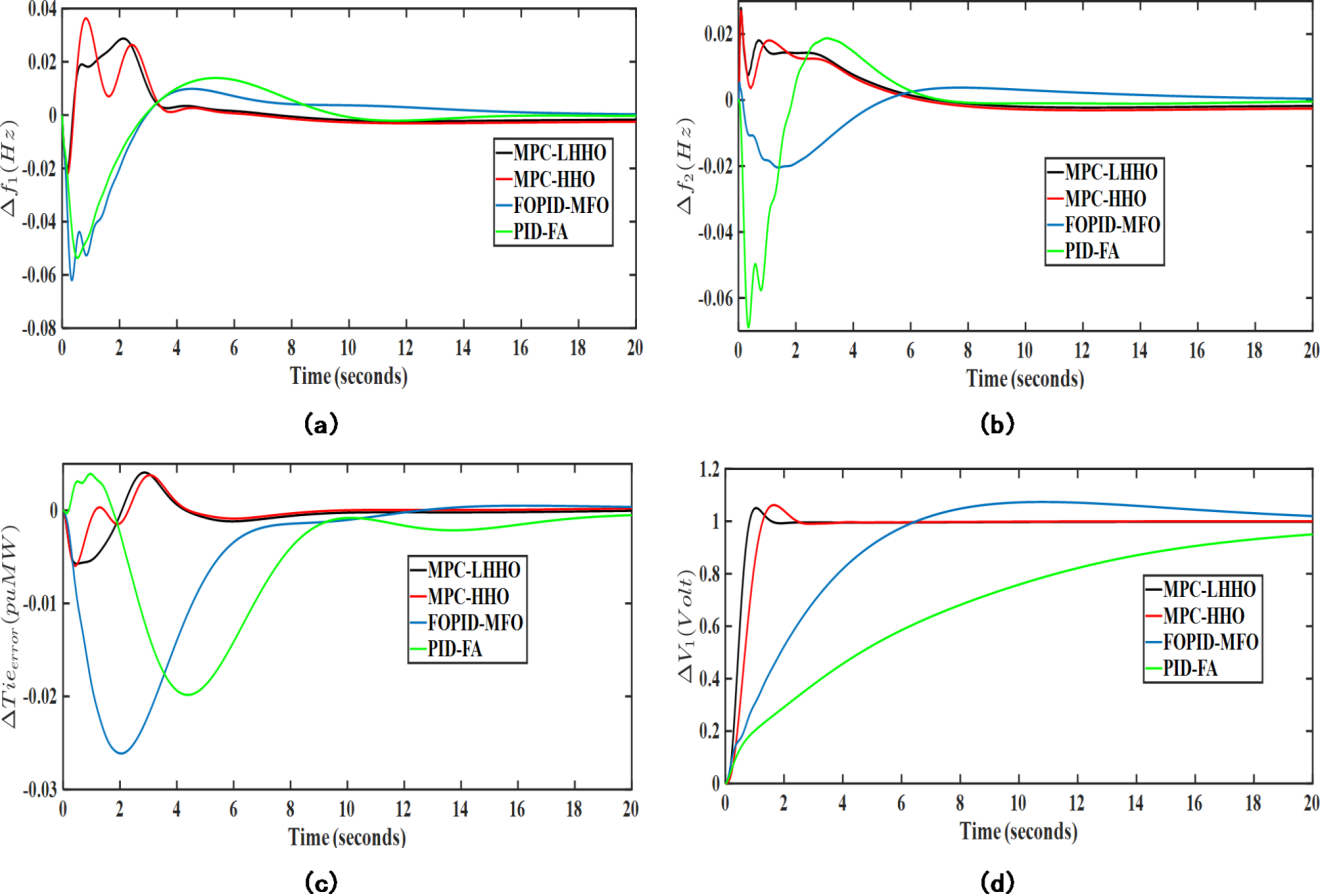
Transient response curves for bilateral transaction case considering; (**a**) $\:\varDelta\:{f}_{1}$ (Hz), (**b**) $\:\varDelta\:{f}_{2}$ (Hz), (**c**) $\:\varDelta\:{T}_{p12}$ (puMW), (**d**) $\:\varDelta\:{V}_{1}$ (V).

Under this analysis, the proposed algorithm was implemented on the combined AVR-LFC system for the presented test system under poolco and bilateral transactions. Herein, the controller performance of the proposed LHHO-based MPC technique is evaluated and compared with the MPC-HHO^29^, PID-FA^27^, and FOPID-MFO^28^ techniques. The controller parameters for the above-mentioned methods are given in Table 1. The resulting graphs showing frequency deviations, tie-line power error, voltage deviations under poolco, and bilateral transaction cases are represented in Figs. 7 and 8, respectively. The quantitative analysis of obtained results from the LHHO-MPC method in comparison with HHO-MPC^29^, PID-FA^27^, and FOPID-MFO^28^ techniques is shown in Table 2. The disco participation matrices for both transactions are given in Eq. (5). The apf values for GENCOs under poolco transaction are [1/3, 1/3, 1/3, 1/3, 1/3, 1/3]; and for bilateral transactions the apf values are [0.5, 0.2, 0.3, 0.4, 0.3, 0.3]. The net amount of generation (in puMW) from each GENCO may be calculated as per the Eq. (6).

5 $$\begin{aligned} & {\text{DPM}}_{{{\text{poolco}}}} = \left[ {\begin{array}{*{20}l} {1/3} \hfill & {1/3} \hfill & {1/3} \hfill & 0 \hfill & 0 \hfill & 0 \hfill \\ {1/3} \hfill & {1/3} \hfill & {1/3} \hfill & 0 \hfill & 0 \hfill & 0 \hfill \\ {1/3} \hfill & {1/3} \hfill & {1/3} \hfill & 0 \hfill & 0 \hfill & 0 \hfill \\ 0 \hfill & 0 \hfill & 0 \hfill & 0 \hfill & 0 \hfill & 0 \hfill \\ 0 \hfill & 0 \hfill & 0 \hfill & 0 \hfill & 0 \hfill & 0 \hfill \\ 0 \hfill & 0 \hfill & 0 \hfill & 0 \hfill & 0 \hfill & 0 \hfill \\ \end{array} } \right]\:{\text{and}} \\ & {\text{DPM}}_{{{\text{bilateral}}}} = \left[ {\begin{array}{*{20}l} {0.2} \hfill & {0.3} \hfill & {0.1} \hfill & {0.2} \hfill & {0.15} \hfill & {0.3} \hfill \\ {0.15} \hfill & {0.1} \hfill & {0.2} \hfill & {0.15} \hfill & {0.15} \hfill & {0.1} \hfill \\ {0.15} \hfill & {0.1} \hfill & {0.2} \hfill & {0.15} \hfill & {0.2} \hfill & {0.1} \hfill \\ {0.3} \hfill & {0.2} \hfill & {0.15} \hfill & {0.1} \hfill & {0.2} \hfill & {0.1} \hfill \\ {0.1} \hfill & {0.2} \hfill & {0.2} \hfill & {0.1} \hfill & {0.1} \hfill & {0.2} \hfill \\ {0.1} \hfill & {0.1} \hfill & {0.15} \hfill & {0.3} \hfill & {0.2} \hfill & {0.2} \hfill \\ \end{array} } \right] \\ \end{aligned}$$

6 $$\:\varDelta\:{P}_{Gi}={\sum\:}_{k=1}^{6}cp{f}_{ik}\varDelta\:{P}_{Lk}$$

Using the above expressions, the net generated power for GENCO 1 under poolco transaction with a load of 0.01 pu MW is calculated as in Eq. (7).

7 $$\:{\Delta\:}{\text{P}}_{\text{G}\text{E}\text{N}\text{C}\text{O}1}=\left[\frac{1}{3}+\frac{1}{3}+\frac{1}{3}+0+0+0\right]\text{*}0.01=\:0.01\:\text{p}\text{u}\text{M}\text{W}$$

Similarly, the net generation for bilateral transaction case is computed as in Eq. (8).

8 $$\:{\Delta\:}{\text{P}}_{\text{G}\text{E}\text{N}\text{C}\text{O}1}=\left[0.2+0.3+0.1+0.2+0.15+0.3\right]\text{*}0.01\:=\:0.0125\:\text{p}\text{u}\text{M}\text{W}$$

**Fig. 9:**
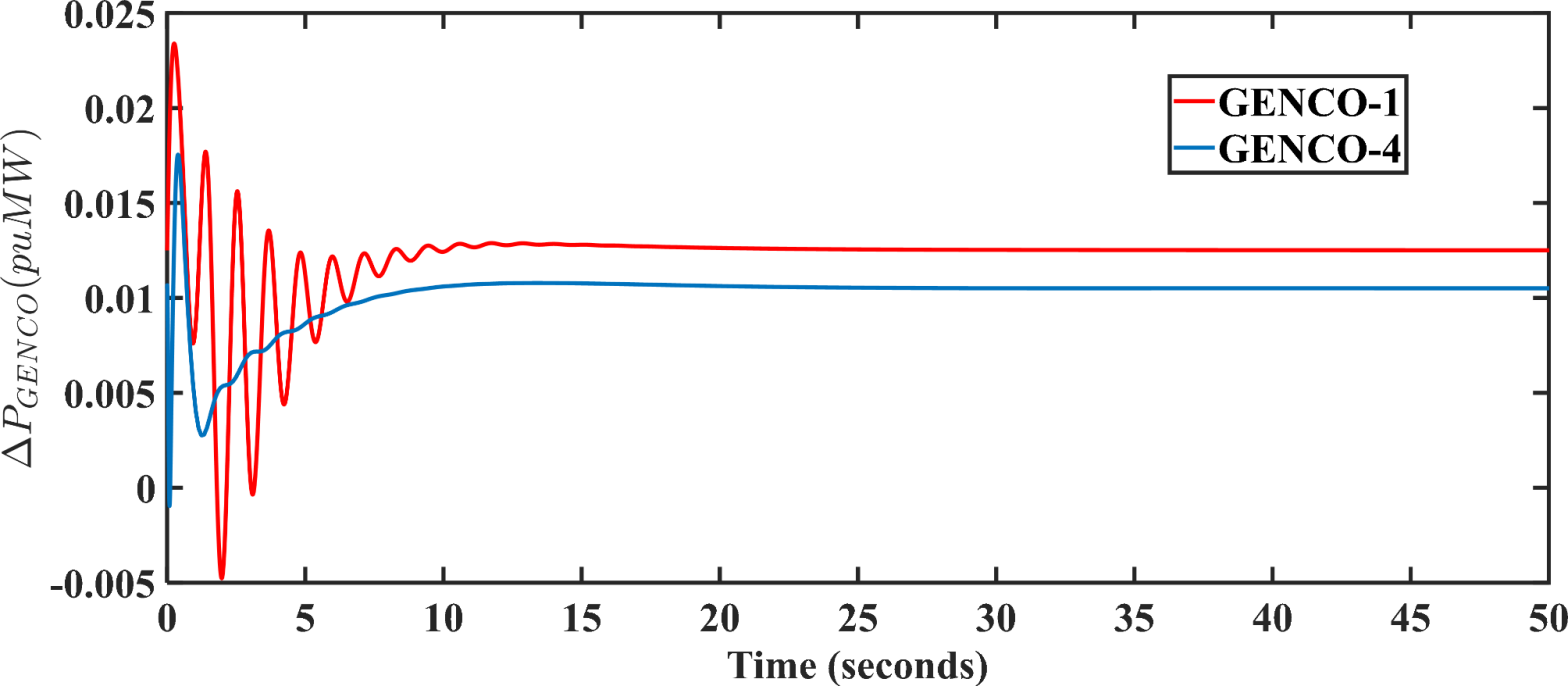
Output power deviations in GENCOs having thermal generation unit.

On the similar lines, the generated output for the remaining GENCOs can be computed using the expression in Eq. (13). For bilateral transaction case, the output for GENCOs 2, 3, 4, 5, and 6 is 0.0085 pu MW, 0.009 pu MW, 0.0105 pu MW, 0.009 pu MW, and 0.0105 pu MW, respectively. For MPC-LHHO controlled system, the generated outputs thermal units in both areas i.e., GENCO 1 and GENCO 4 under bilateral transaction case are provided in Fig. 9.

Table 3 depicts the transient response analysis of obtained results in terms of peak overshoot (PO), peak undershoot (PU), and settling time (ST). Upon analyzing the same, it has been observed that for the stabilization frequency in area 1 under poolco transactions, the proposed MPC-LHHO method provides a minimum undershoot of 0.0207, which is 67.45% and 62.83% improved when compared with PID-FA^27^ and FOPID-MFO^28^ controllers, respectively. Similarly, the presented method provides 91.11% and 84% improvements in settling time for voltage deviations in area 1 under poolco transactions when compared to the PID-FA^27^ and FOPID-MFO^28^ controllers, respectively. The proposed MPC-LHHO method provides a small yet significant improvement in overall fitness function values when compared to the MPC-HHO^29^ controller.

**Table 3:** Transient response performance evaluation for different controllers.

Type	Controller		PID-FA^27^	FOPID-MFO^28^	MPC-HHO^29^	MPC-LHHO (proposed)
Poolco transaction	PI	ISE_1_	0.0069	0.0068	0.0015	**0.0011**
		ISE_2_	2.5358	2.1525	0.9036	**0.8524**
	$\:\varDelta\:{f}_{1}$	PO	0.0136	0.0111	0.0305	0.0292
		PU	− 0.0636	− 0.0557	− 0.0207	− 0.0207
		ST	14 s	20.5 s	8.5 s	8.5 s
	$\:\varDelta\:{f}_{2}$	PO	0.0034	0.0046	0.0114	0.0048
		PU	− 0.0322	− 0.0286	− 0.0242	− 0.0131
		ST	19 s	19 s	13 s	12 s
	$\:\varDelta\:{T}_{p12}$	PO	0.0012	0.0033	0.0152	0.0112
		PU	− 0.0214	− 0.0209	− 0.0044	− 0.0036
		ST	16 s	16 s	11 s	11 s
	$\:\varDelta\:{V}_{1}$	PO	0.0	1.049	1.07	1.046
		PU	0.0	0.0	0.0	0.0
		ST	22.5 s	12.5 s	2.5 s	2 s
Bilateral transaction	PI	ISE_1_	0.0083	0.0066	0.0032	**0.0027**
		ISE_2_	3.3465	2.5132	0.7561	**0.6036**
	$\:\varDelta\:{f}_{1}$	PO	0.0138	0.0098	0.0219	0.0287
		PU	− 0.0534	− 0.0612	− 0.0363	− 0.0212
		ST	25.5 s	20 s	22 s	19 s
	$\:\varDelta\:{f}_{2}$	PO	0.0187	0.0051	0.0269	0.0279
		PU	− 0.0689	− 0.0203	− 0.003	0.0
		ST	29.0 s	24 s	27 s	16 s
	$\:\varDelta\:{T}_{p12}$	PO	0.0039	0.0	0.0037	0.0057
		PU	− 0.0198	− 0.0265	− 0.0059	− 0.00405
		ST	30.0 s	25 s	20 s	19 s
	$\:\varDelta\:{V}_{1}$	PO	0.0	1.075	1.042	1.041
		PU	0.0	0.0	0.0	0.0
		ST	25.5 s	20.5 s	2.5 s	1.5 s

Significant values are in bold.

Further, considering bilateral transactions case, the proposed MPC-LHHO method yields minimum value of overall fitness function value considering LFC loops (ISE_1_), i.e., 0.0027. Comparatively, the proposed technique results in 67.46%, 59.09%, and 15.62% improvements in LFC loops (ISE_1_) values when compared with PID-FA^27^, FOPID-MFO^28^, and MPC-HHO^29^ controllers, respectively. Further, the fitness function value corresponding to the AVR loop is minimum for MPC-LHHO controller, i.e., 0.6036. Herein, an improvement of 81.96%, 75.98%, and 20.16% has been observed when compared with PID-FA^27^, FOPID-MFO^28^, and MPC-HHO^29^ controllers, respectively.

### Performance improvement using UPFC and EV

**Fig. 10:**
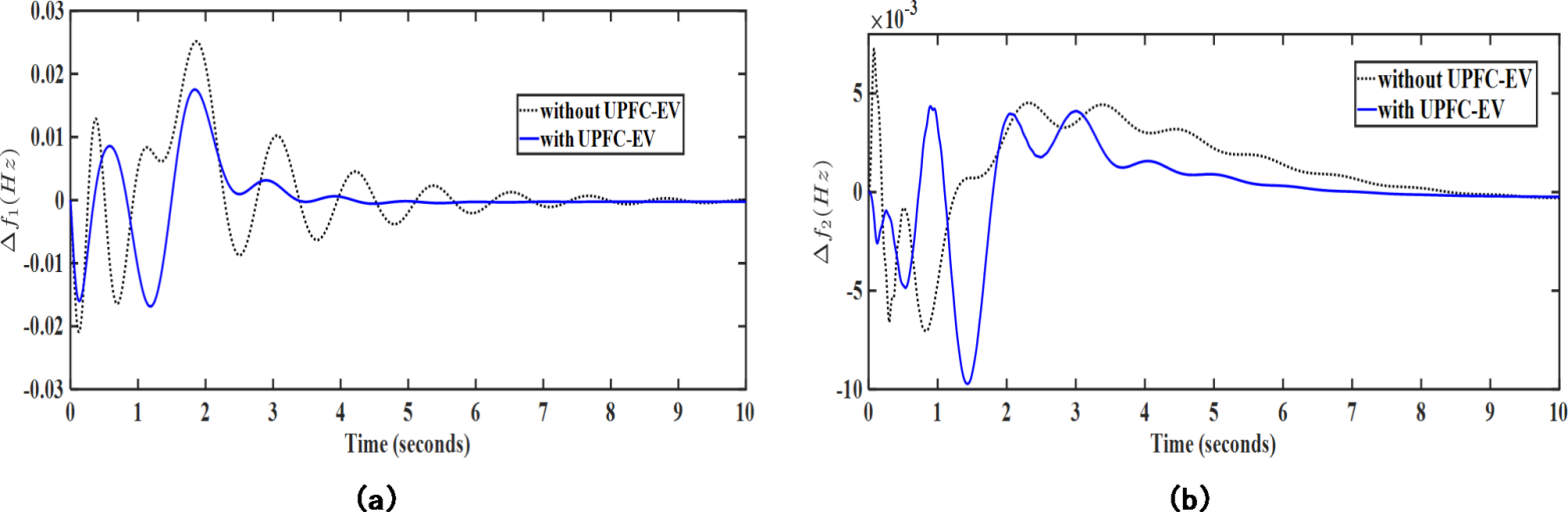
Graphical representation of transient response performance obtained after using UPFC and EV integration for poolco transaction case; (**a**) $\:\varDelta\:{f}_{1}$ (Hz), (**b**) $\:\varDelta\:{f}_{2}$ (Hz).

**Fig. 11:**
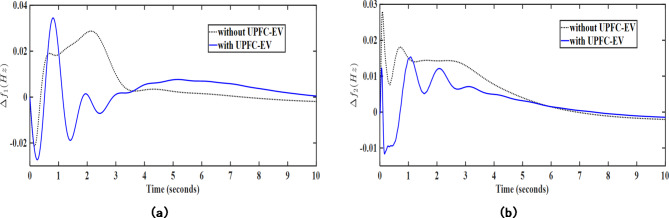
Graphical representation of transient response performance obtained after using UPFC and EV integration for bilateral transaction case; (**a**) $\:\varDelta\:{f}_{1}$ (Hz), (**b**) $\:\varDelta\:{f}_{2}$ (Hz).

Herein, the load frequency controller performance has been further enhanced with the aid of certain auxiliary controller loops working in coordination with the proposed MPC-LHHO controller. Herein, UPFC device has been used to provide supplementary support to provide enhanced power exchange between the two control areas. Thereafter, the electric vehicle integration with the grid has been considered along with UPFC device and the MPC weights have been recomputed in coordination with these two auxiliary LFC loops. From Figs. 10 and 11, it is evident that these auxiliary loops significantly reduce the frequency deviations in the system under poolco and bilateral transaction cases. Here, the fitness function values corresponding to the frequency deviation in both cases of the MPC-LHHO controlled system aided with UPFC and EV loops demonstrate the improvement in overall performance enhancement in the frequency regulation.

### Performance evaluation under stochastic modelling of RESs

This section features the study and implementation of stochastic wind and solar models in EV-UPFC assisted MPC-LHHO controlled AVR-LFC system under poolco transactions. Herein, the two different cases for the stochastic models have been considered for the transient analysis.

**Fig. 12:**
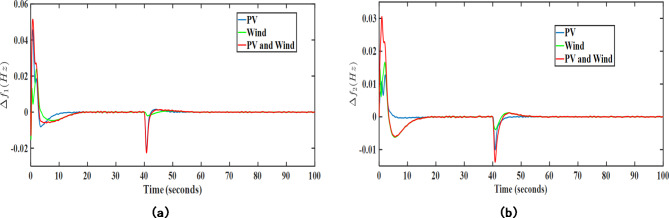
Obtained transient response for SM-1 case; (**a**) $\:\varDelta\:{f}_{1}$ (Hz), (**b**) $\:\varDelta\:{f}_{2}$ (Hz).

In the first case (SM-1), the wind and solar generation values have been modelled considering deterministic shift and stochastic drifts. Further, the proposed EV-UPFC assisted MPC-LHHO controller has been applied with original weight settings as discussed in previous subsections. The resulting transient response plots have been acquired and represented in Fig. 12. From the obtained plots, it is clear that the frequency and voltage profile of the system remain stable under the stochastic modelling (SM-1) of wind and solar power generation.

**Fig. 13:**
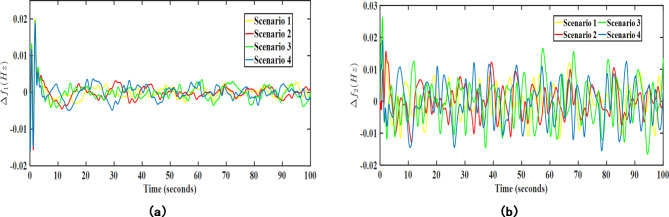
Transient response performance results under SM-2 case considering wind turbine in area 2; (**a**) $\:\varDelta\:{f}_{1}$ (Hz), (**b**) $\:\varDelta\:{f}_{2}$ (Hz).

**Fig. 14:**
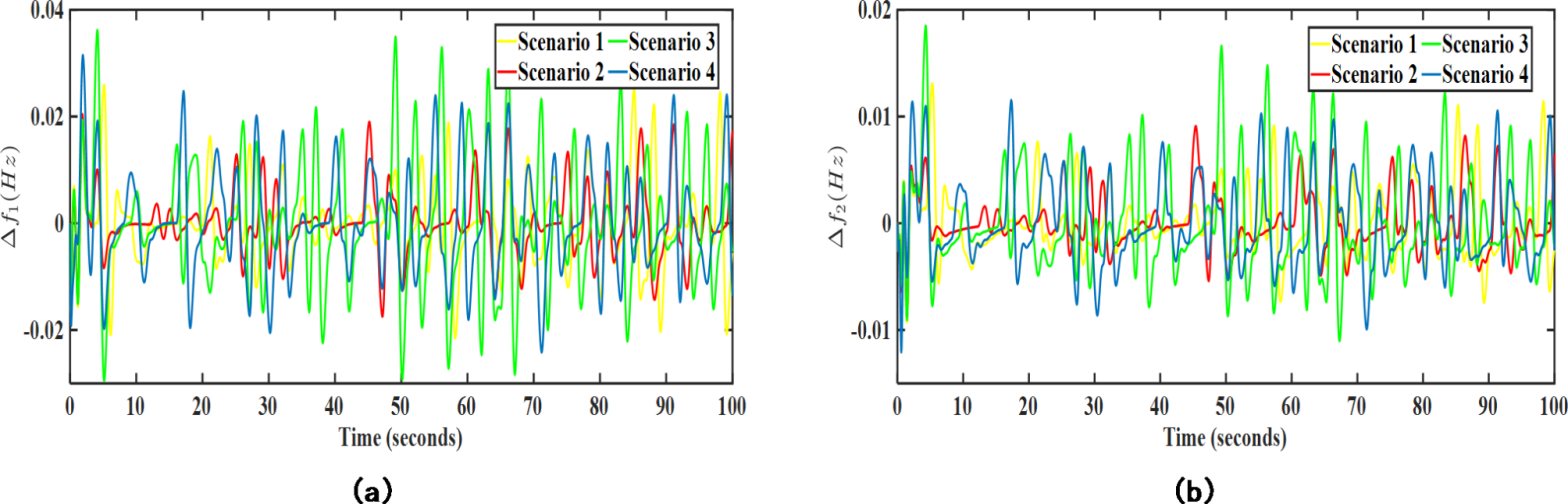
Transient response performance results under SM-2 case considering solar PV integration in area 1; (**a**) $\:\varDelta\:{f}_{1}$ (Hz), (**b**) $\:\varDelta\:{f}_{2}$ (Hz).

In the second case (SM-2), the randomness in wind and solar data has been modelled with the assistance of Monte Carlo simulated ARIMA models of real time wind speed and solar irradiance data. Further, these stochastic models of PV and wind have been added in the system in addition to the existing transfer function-based PV and wind models in area 1 and area 2, respectively. The resulting transient response plots attained after applying SM-2 modelling are given in Figs. 13 and 14.

### Performance assessment under diverse loading cases

**Fig. 15:**
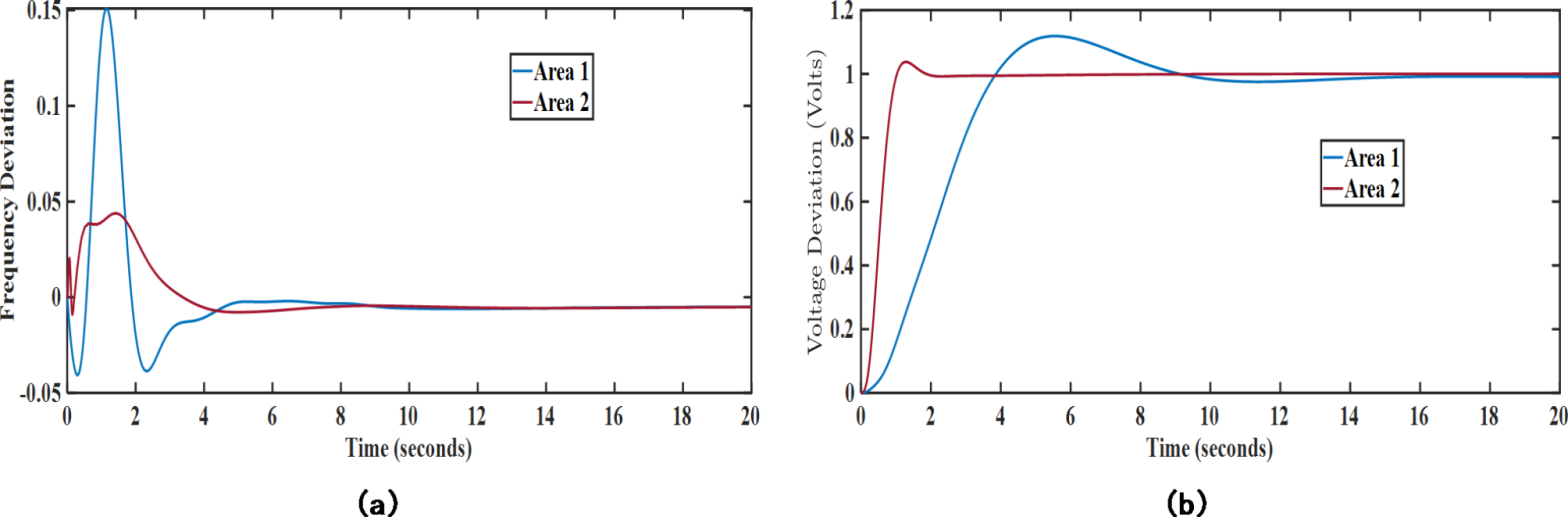
Transient response results under contract violation case.

In this analysis, the robustness of the presented approach has been tested under different challenging scenarios such as contract violation and random loading cases. The proposed UPFC-EV assisted MPC-LHHO approach has been implemented after considering 0.01 pu MW additional load demand from DISCO 1. The DPM for the contract violation case is taken from^24^, and the apf values for the GENCOs are given as [0.4, 0.25, 0.35, 0.4, 0.2, 0.4]. The resultant response of the system frequency and voltage profile is given in Fig. 15.

**Fig. 16:**
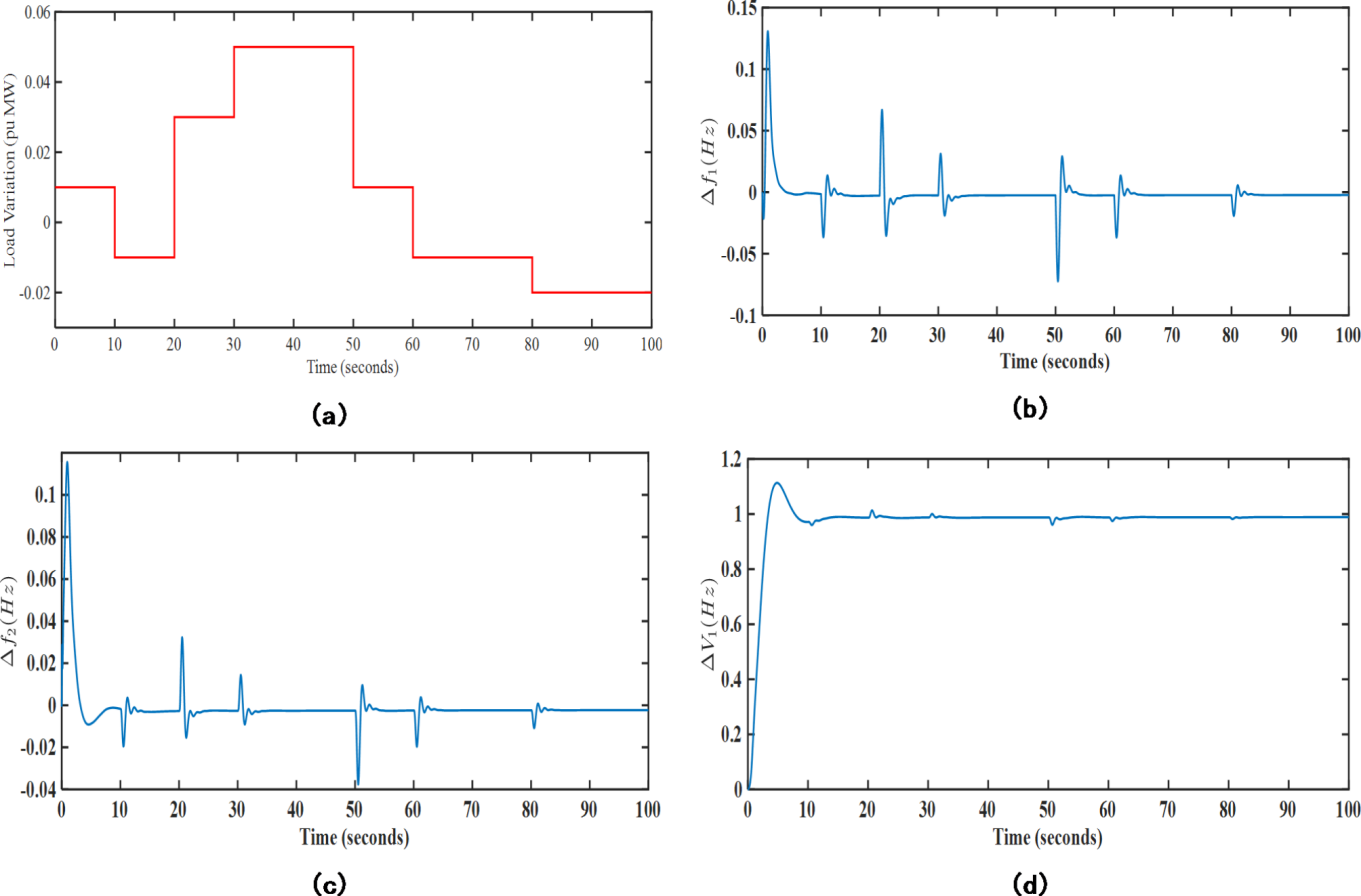
Transient response curves for random loading case; (**a**) Load (pu MW), (**b**) $\:\varDelta\:{f}_{1}$ (Hz), (**c**) $\:\varDelta\:{f}_{2}$ (Hz), (**d**) $\:\varDelta\:{V}_{1}$ (V).

Further, the robustness of the proposed UPFC-EV based MPC-LHHO controller has been tested under the random loading scenario. Herein, a randomly varying load pattern has been considered in the contract violation case. From the transient response plots in Fig. 16, it is clear that the proposed controller effectively handles the fluctuating loading pattern to give a stable transient response for frequency and terminal voltage errors.

## Conclusions

This manuscript addresses the dual challenge of reducing voltage and frequency deviations in a deregulated power network that includes thermal, diesel, and renewable sources from wind, solar PV, and hydro plants. An efficient control strategy, the Leader Harris Hawks optimized Model Predictive Control (MPC-LHHO), is implemented to stabilize voltage and frequency in this hybrid interconnected network, specifically under poolco and bilateral transaction scenarios. Simulation results confirm the superiority of this approach, as it achieves minimal fitness function values and enhances the system’s transient profile compared to other recent methods reported in the literature. For both transaction cases, the MPC-LHHO controller demonstrates outstanding performance, yielding the lowest performance index values. Additionally, an auxiliary control loop involving unified power flow controller and electric vehicle (UPFC-EV) integration is incorporated with the MPC-LHHO to further refine frequency stability, with EV-UPFC integration boosting the fitness function value by 52.18% and 22.22% for poolco and bilateral scenarios, respectively. To address practical considerations, two separate methods are used to incorporate stochastic variations in solar radiation and wind speed into the voltage and frequency control model. Results indicate the robustness of the proposed control method, delivering stable responses across various load conditions, including contract violations and random loading scenarios.

Future works may focus on integrating the proposed control strategy with real-time market dynamics and testing it in deregulated power systems. Additionally, expanding the stochastic models to account for more uncertainty factors, such as weather and market fluctuations, could improve the predictive capability. Exploring hybrid optimization techniques and addressing cybersecurity concerns will also be crucial for enhancing the robustness and adaptability of the system in future implementations.

## Data Availability

The datasets associated with the current study will be available from the corresponding author on request.

## Appendix

*i =* 1, and 2. T = 50 s. a_12_ = -0.25, *Thermal*: $\:{T}_{g}$= 0.08 s; $\:{T}_{t}$= 0.3 s; $\:{T}_{r}$= 10 s; $\:{K}_{r}=0.5.$*Wind*: $\:{T}_{w1}$= 0.6 s; $\:{T}_{w2}$ = 0.041 s; $\:{K}_{w1}$ = 1.25; $\:{K}_{w2}$ = 1.4; $\:BC=1$. *Diesel*: $\:{K}_{dielsei}$= 16.5. *PV plant*: *a*_*pv*_ =900; *b*_*pv*_ = -18; *c*_*pv*_ = 100; *d*_*pv*_ =50. *Hydro*: $\:{T}_{RH}$=48.7 s; $\:{T}_{R}$=5 s; $\:{T}_{GH}$=0.513 s; $\:{T}_{w}$= 1 s. $\:{T}_{ij}=$ 0.08 pu. *R*= 2.4 Hz/puMW. B= 0.425 puMW/Hz. $\:{K}_{pi}$= 120 Hz/pu MW. $\:{T}_{pi}$= 20 s. SM-1 *(Wind)*: $\:\eta\:$ = 0.8; $\:\beta\:$ = 10; $\:\partial\:$= 1. SM-1 (*Solar)*: $\:\eta\:$ = 0.7; $\:\beta\:$ = 2; $\:\partial\:$= 0.1. SM-2 (*Solar)*: $\:\eta\:$ = 10%; S= 4048m^2^, *T*_a_=27.8^o^C. *EV Model*: $\:{K}_{EVi}$*=* 1; $\:{T}_{EVi}$ = 1; $\:{R}_{EVi}$ = 2.4.
